# Choice of Aspiration Prevention Surgery for Patients With Neuromuscular Disorders: Report of Three Cases

**DOI:** 10.3389/fsurg.2019.00066

**Published:** 2019-11-21

**Authors:** Mitsuhiko Katoh, Rumi Ueha, Taku Sato, Shunichi Sugasawa, Takao Goto, Akihito Yamauchi, Tatsuya Yamasoba

**Affiliations:** Department of Otolaryngology, The University of Tokyo, Tokyo, Japan

**Keywords:** aspiration prevention surgery, neuromuscular disorders, upper esophageal sphincter opening during swallowing, dysphagia, quality of life

## Abstract

Dysphagia, one of the major complications of neuromuscular diseases such as Parkinson's disease and amyotrophic lateral sclerosis (ALS), decreases quality of life and may lead to malnutrition or aspiration pneumonia. Although recent reports have suggested that surgical aspiration prevention improves quality of life and enables oral intake, the selection of appropriate aspiration prevention techniques has rarely been discussed. In this report, we present the cases of three patients with neuromuscular diseases who underwent surgical aspiration prevention; we selected the surgical techniques based on analysis of the dysphagia mechanisms, disease progression, and general condition in each case. Case 1 was a 55-year-old man with multiple system atrophy (MSA) and presented with dysphagia associated with insufficient upper esophageal sphincter (UES) relaxation. We performed central-part laryngectomy, which was able to improve UES relaxation. Case 2 was a 79-year-old man with progressive supranuclear palsy who presented with respiratory disorder and dysphagia. Glottic closure under local anesthesia was selected because he also had acute hepatobiliary dysfunction and methicillin-resistant *Staphylococcus aureus* pneumonia with pleural effusion. Case 3 was a 75-year-old man with ALS and presented with respiratory disorder and mild dysphagia. Subglottic closure with total cricoidectomy was selected because his dysphagia was expected to worsen due to tracheostomy and disease progression. We also summarize the characteristics of the aspiration prevention surgical techniques based on our cases and on literature review. The causes of dysphagia, including insufficient UES opening during swallowing, weak pharyngeal constriction, velopharyngeal insufficiency, and inadequate laryngeal elevation, should be assessed by detailed examination before surgery, and the type of aspiration prevention surgery should be selected based on patient swallowing function and general condition.

## Introduction

Dysphagia is an important complication in patients with neuromuscular disorders, including Parkinson's disease and amyotrophic lateral sclerosis (ALS); it negatively impacts patient quality of life and may lead to fatal outcome due to malnutrition and aspiration pneumonia (1, 2). It is reported that there are various underlying mechanisms of dysphagia in neuromuscular diseases: deficient relaxation of the upper esophageal sphincter (UES) during swallowing in patients with Parkinson's disease (3), abnormal deglutitive proximal esophageal contraction and insufficient UES opening in patients with multiple system atrophy (MSA) (4), and weakness of pharyngeal contraction and impaired laryngeal elevation in patients with ALS (5). It has been reported that aspiration prevention surgery for severe dysphagia can improve the quality of life of patients and their families and help patients regain oral intake (6–8). There are several surgical techniques for aspiration prevention, such as total laryngectomy, central-part laryngectomy, tracheoesophageal diversion, laryngotracheal separation, and laryngeal closure (7, 9–12). Total laryngectomy is performed under general anesthesia and is highly invasive with longer operation time and larger amount of bleeding compared with other procedures (13, 14). Central-part laryngectomy is conducted under general anesthesia and it is less invasive compared to total laryngectomy (9). Laryngotracheal separation and diversion have been performed since the 1970s, and these techniques can take place under both general and local anesthesia (10, 11). Laryngeal closure can be performed under local anesthesia (7, 15). Considering that the mechanism of dysphagia differs among patients depending on the neuromuscular disorder (1), selecting an appropriate surgical procedure for aspiration prevention followed by proper assessment of the dysphagia pattern in each patient is essential. However, there has been no detailed information on the selection of surgical procedure in accordance to patient condition and swallowing function in the literature. Herein, we present the cases of three patients with neuromuscular disorders with favorable outcome after surgical treatment for dysphagia and discuss the appropriate selection of aspiration prevention surgery for patients with neuromuscular disorders depending on patient background, including the swallowing pattern, physical condition, and disease progression.

## Case Reports

### Case 1

A 55-year-old man developed dysarthria at age 50 years and gait disorder at age 51 years. He was diagnosed with MSA at age 53 years. At the age of 55 years, he was still able to orally intake nourishment and walk with assistance, but dyspnea suddenly appeared after an incident of vomiting. He was transferred to the Neurological Department of our institution by ambulance and developed respiratory disorder due to aspiration pneumonia. Although general treatment under tracheal intubation was performed for 1 week, extubation was considered difficult due to the large amount of aspirated saliva and sputum in the respiratory tract. Hence, otolaryngologists were consulted for the possibility of tracheostomy. The results of the blood tests were as follows: albumin, 3.0 g/dL; C-reactive protein, 3.04 mg/dL; white blood cell count, 13,800/μL; neutrophils, 77.5%; eosinophils, 2.5%; basophils, 0.5%; monocytes, 4.7%; and lymphocytes, 14.8%. Blood gas analysis revealed a pH of 7.45, carbon dioxide partial pressure of 41.1 mm Hg, and oxygen partial pressure of 134 mmHg (FiO_2_ 50%).

**Figure 1:**
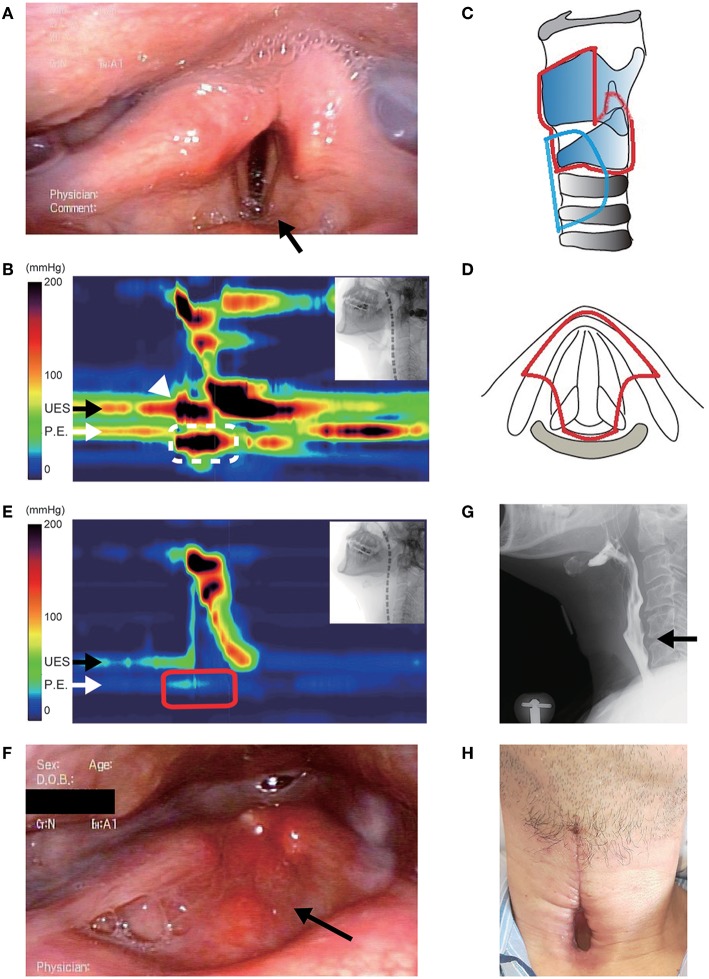
Clinical findings in case 1. **(A)** Preoperative fiberoscopic view. Vocal cord abduction was insufficient bilaterally, and saliva was aspirated into the subglottic area (arrow). **(B)** Preoperative high-resolution manofluorography (HRMF) finding. The black arrow shows the level of the upper esophageal sphincter (UES). The white arrow shows the level of the proximal esophagus (PE). HRMF revealed UES opening impairment during swallowing (white arrowhead) and abnormal deglutitive proximal esophageal contraction (ADPEC, the area surrounded by a white broken line). **(C,D)** Schemas of the surgery. Lateral view **(C)** and axial view **(D)**. The removed area is encircled by a red line, and the location of the permanent tracheostoma is encircled by a blue line. **(E)** Postoperative HRMF finding. The resting UES pressure became low, and ADPEC disappeared postoperatively (the area surrounded by a red line). **(F)** Laryngeal fiberoscopic view after the surgery. The supralaryngeal mucosal surface appears smooth (arrow). **(G)** The videofluoroscopic swallowing study showed laryngeal closure without leakage and sufficient UES opening (arrow). **(H)** Permanent tracheostoma after surgery.

Even after the tracheostomy, the patient had difficulty in swallowing saliva, resulting in discharge of copious amounts of sputum from the tracheostomy tube and the suction lumen. Laryngeal fiberoscopy showed bilateral vocal cord abduction impairment (Figure 1A). Videoendoscopic evaluation of swallowing revealed severe oropharyngeal dysphagia. The videofluoroscopic swallowing study (VFSS) showed no apparent impairment in the oral phase of swallowing, although mild dysarthria was observed. High-resolution manofluorography (HRMF) showed deficient relaxation of the UES during swallowing and abnormal deglutitive proximal esophageal contraction (Figure 1B) (4). To secure an air way and maintain it clear, aspiration prevention surgery was considered. Surgery under general anesthesia was scheduled, as the patient strongly refused surgery under local anesthesia and his general condition was relatively good.

Reduction of the abnormally high pressure at the UES was considered important for smooth pharyngoesophageal passage in this patient. As cricopharyngeal myotomy appeared to insufficiently contribute to the improvement of UES opening during swallowing due to physical obstruction by the remaining lamina of the cricoid cartilage, modified central-part laryngectomy (9), which is less invasive and reduces the pressure of the UES by removing the whole cricoid cartilage, was performed (Figures 1C,D). Intraoperative bleeding was limited, and the operation lasted 151 min. Postoperatively, VFSS showed a wide upper esophageal opening and smooth bolus passage from the pharynx to the cervical esophagus without leakage at the surgical site (Figure 1E). HRMF on day 11 postoperatively revealed hypotensive resting UES pressure and disappearance of abnormal deglutitive proximal esophageal contraction (Figure 1F). The patient was able to restart oral intake 13 days postoperatively, and the tracheal cannula could be removed from his tracheostoma (Figure 1G). One month postoperatively, the mucosal surface of the closure site at the supraglottic area became smooth (Figure 1H), and the patient could enjoy regular oral food intake.

### Case 2

A 79-year-old man developed parkinsonism with bradykinesia at age 74 years and was diagnosed with progressive supranuclear palsy at the age of 78 years. At the age of 79 years, he became bedridden and developed speech disorder, and a gastrostomy was created in place of oral feeding for prevention of recurrent aspiration pneumonia. As the difficulty in sputum expectoration and respiratory disorder progressed, a nasal airway tube was inserted to suction pharyngeal phlegm and maintain the airway from the nose to the larynx. However, the respiratory disorder progressively became severe, and the patient was transferred to our hospital for a tracheostomy.

**Figure 2:**
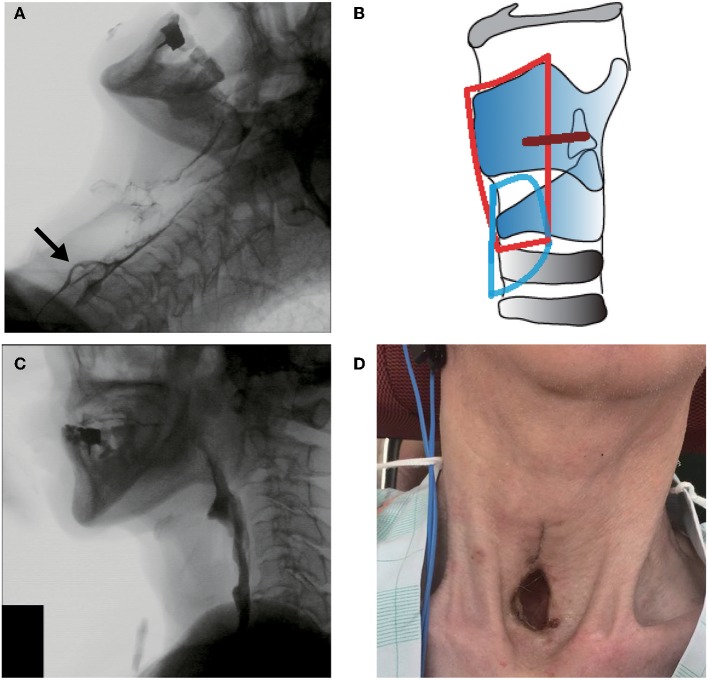
Clinical findings in case 2. **(A)** Preoperative videofluoroscopic swallowing study (VFSS). The arrow shows contrast agent aspiration into the trachea during swallowing. **(B)** Operative schema. The removed area is encircled by a red line, and the place of the permanent tracheostoma is encircled by a blue line. The glottic closure site is shown as a brown line. **(C)** Postoperative VFSS. **(D)** View of the permanent tracheostoma without a cannula.

At the time of admission, acute exacerbation of chronic cholecystitis and pneumonia with pleural effusion occurred, and the following blood test results were obtained: aspartate aminotransferase, 88 IU/L; alanine aminotransferase, 102 IU/L; lactate dehydrogenase, 200 IU/L; γ-glutamyl transpeptidase, 546 IU/L; alkaline phosphatase, 1,453 IU/L; blood urea nitrogen, 12 mg/dL; creatinine, 0.50 mg/dL; sodium, 141 mEq/L; potassium, 4.3 mEq/L; chloride, 100 mEq/L; albumin, 2.7 g/dL; C-reactive protein, 1.52 mg/dL; white blood cell count, 3,060/μL; neutrophils, 50.0%; eosinophils, 1.0%; basophils, 1.0%; monocytes, 4.0%; and lymphocytes, 43.0%. Blood gas analysis revealed a pH of 7.49, carbon dioxide partial pressure of 47.6 mm Hg, and oxygen partial pressure of 77 mmHg (room air). Chest radiography and chest computed tomography revealed bilateral pleural effusions. Methicillin-resistant *Staphylococcus aureus* was detected in the sputum culture. Laryngoscopy showed normal vocal cord movement, saliva pooling in the hypopharynx, and saliva penetration into the larynx. VFSS showed deteriorated pharyngeal contraction, impaired laryngeal elevation, and silent aspiration without clearance from the airway (Figure 2A).

The patient was diagnosed with severe oropharyngeal dysphagia related to respiratory impairment, and then aspiration prevention surgery and permanent-tracheostoma creation were proposed to prevent airway obstruction and aspiration pneumonia, which are common causes of progressive supranuclear palsy death (16). Considering his poor general condition with acute hepatobiliary function impairment and methicillin-resistant *Staphylococcus aureus* pneumonia with pleural effusion, glottic closure under local anesthesia was performed and a large permanent tracheostoma was created (Figure 2B). Intraoperative bleeding amounted to <5 mL, and the operation lasted 120 min. As VFSS on day 11 postoperatively showed no leakage at the surgical site (Figure 2C), he could restart oral liquid intake. Although solid food intake was difficult due to the impaired oral phase of swallowing and poor food passage at the UES portion, the frequency of secretion suctioning was dramatically decreased postoperatively, and the patient was discharged without a tracheal cannula (Figure 2D).

### Case 3

A 75-year-old man had experienced stumbling while walking and difficulty in arm raising at age 73 years. He was diagnosed with flail arm ALS and developed respiratory disorder at the age of 74 years, for which treatment with non-invasive positive pressure ventilation started at that time. As mechanical ventilatory support was required due to deterioration of respiratory function after pneumonia, the neurologists considered tracheostomy and/or surgical aspiration prevention, which is recommended in the Japanese guidelines if patients wish to intake food orally (17), and otolaryngologists were consulted.

**Figure 3:**
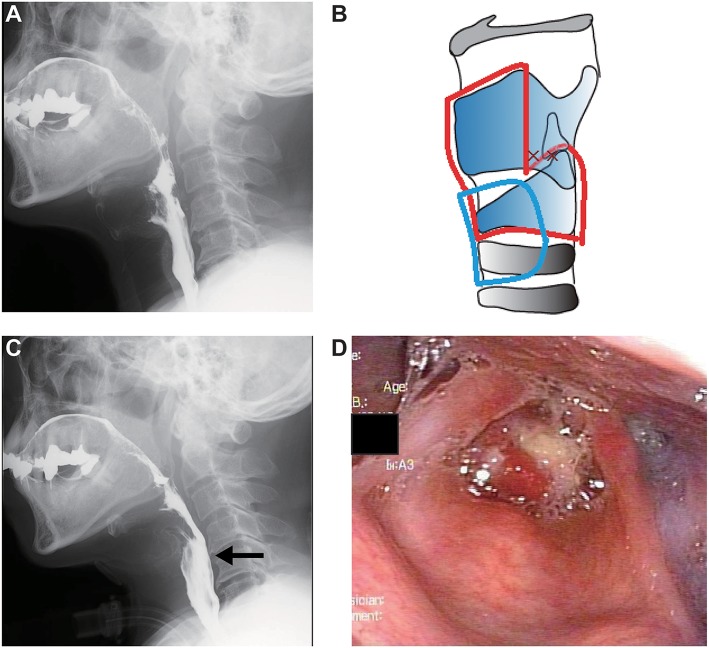
Clinical findings in case 3. **(A)** Preoperative videofluoroscopic swallowing study (VFSS). **(B)** Operative schema. The removed area is encircled by a red line, and the permanent tracheostoma is located in the area surrounded by a blue line. **(C)** Postoperative VFSS. The arrow shows sufficient upper esophageal sphincter opening. **(D)** Postoperative laryngeal fiberoscopic view.

At the first visit at our department, he was wheelchair bound, used non-invasive positive pressure ventilation, and could eat normally with a little difficulty. The blood tests revealed no considerable issues. Blood gas analysis revealed a pH of 7.386, carbon dioxide partial pressure of 50.7 mm Hg, and oxygen partial pressure of 81.8 mm Hg (2 L/min of oxygen). Vital capacity was 2.18 L (%vital capacity, 58.0%) as shown by spirometry tests. VFSS revealed that there was no obvious aspiration during liquid swallowing, except for short duration of velopharyngeal closure and small volumes of pharyngeal residue (Figure 3A).

Tracheostomy and simultaneous aspiration prevention surgery were planned because the swallowing function was expected to worsen with the tracheostomy and with ALS progression and the patient strongly desired to be treated with aspiration prevention surgery. Subglottic closure with total cricoidectomy (Figure 3B) was performed under general anesthesia to shorten the operation duration and avoid postoperative pharyngoesophageal passing disturbance due to the tracheal cannula burden and the artificial ventilation tube. Intraoperative bleeding was limited, and the operation lasted 125 min. VFSS and laryngofibroscopy on day 13 postoperatively showed no leakage or infection at the surgical site (Figures 3C,D). The patient could restart normal oral food intake despite using mechanical ventilation via the tracheostomy.

## Discussion

We presented the cases of three patients with neuromuscular disorders who underwent aspiration prevention surgery that was individually selected for each patient. To select a proper surgical procedure for aspiration prevention, we preoperatively attempt to perform detailed examinations for swallowing function including VFSS, HRMF (if possible), and physical examinations such as cardiac or respiratory studies. We believe that the appropriate surgical procedures for aspiration prevention should be selected with consideration of the patient's physical status, prognosis, expected time course, and the characteristics of the dysphagia such as impaired UES relaxation during swallowing.

Patients with neuromuscular disorders, such as Parkinson's disease, MSA, and ALS often develop dysphagia as the diseases progress (1, 2, 18). The patterns of dysphagia, such as oral dysfunction, pharyngeal dysfunction, and esophageal dysfunction, vary depending on the disease. In cases of pharyngeal dysphagia, impaired UES relaxation often occurs in Parkinson's disease and MSA (3, 7). Inadequate duration of UES relaxation and abnormal hyper-pressure of the resting UES are often recognized in patients with MSA regardless of the MSA type (4, 7). Impaired laryngeal elevation and reduced pharyngeal contraction can often be observed in patients with ALS resulting from weakness of the pharyngeal muscles with disease progression (5). Inadequate laryngeal elevation and impaired pharyngeal contraction can result in physically diminished UES opening, occluding the passage from the pharynx to the esophagus. In cases of patients with neuromuscular disorders who require aspiration prevention and have impaired UES opening or the potential for its development, surgical techniques such as cricopharyngeal myotomy (19) and cricoidectomy (20), which facilitate bolus passage of the UES section, should be performed simultaneously with aspiration prevention surgery, as only aspiration prevention supposedly cannot sufficiently improve UES passage. In case 1, as impaired UES relaxation during swallowing was detected by HRMF, we selected central-part laryngectomy to prevent aspiration and improve UES opening during swallowing. In case 3, we performed total cricoidectomy at the same time with subglottic closure considering the slightly impaired velopharyngeal closure and ALS progression. Given that oropharyngeal swallowing function in patients with bulbar-onset ALS can deteriorate earlier than that in patients with other types of ALS, aspiration prevention surgery should be considered for patients with bulbar-onset ALS.

Respiratory dysfunction is also caused by neuromuscular disorders, and tracheostomy is needed to manage artificial ventilation. As is well-known, tracheostomy can impair swallowing function (21), and thus deterioration of swallowing after tracheostomy should be considered for patients with artificial ventilation. In case 3, we conducted subglottic closure with total cricoidectomy because the swallowing function, including the UES opening, was expected to worsen with tracheostomy and disease progression.

Recently, aspiration prevention surgery has been considered to be a better option for postoperative feeding and quality of life in patients with irreversible severe dysphagia and impaired articulation or vocal function in comparison with only tracheostomy (7, 22). However, few reports have focused on appropriate selection of aspiration prevention techniques. The characteristics of aspiration prevention surgeries are summarized in Table 1. It is essential to consider the surgical stress of aspiration prevention including anesthesia, bleeding, and operation time because the general condition of patients undergoing aspiration prevention is often poor due to their underlying disease. Total laryngectomy is highly invasive because of general anesthesia, longer operation time, and larger amount of bleeding compared with other techniques (13, 14). Central-part laryngectomy is less invasive compared to total laryngectomy because operation time is shorter and bleeding is limited (9). Laryngotracheal separation and diversion are also less invasive because these techniques can be performed under both general and local anesthesia and bleeding is limited and operation time is shorter than those in total laryngectomy (10, 11). Laryngeal closure, including glottic and subglottic closure, is less invasive compared to laryngotracheal separation and diversion because it can be performed under local anesthesia within an average of approximately 120 min (7, 15). In addition to surgical stress, it is important to consider the surgical effect on the UES because insufficient UES relaxation also disturbs swallowing function as seen in case 1. Total and central-part laryngectomy can result in UES opening, and subglottic closure with total cricoidectomy also enables bolus passage at the UES portion easily as in case 3. In addition, it is important to consider the general status of the patient, including cardiac and respiratory function, when selecting the appropriate surgical procedure for aspiration prevention; surgery must take place under local anesthesia when the patient's general condition is poor as in case 2. Aspiration prevention eliminates the possibility of aspiration pneumonia and airway obstruction and can prolong life expectancy. Thus, quality of life can be further improved by appropriate choice of surgical techniques considering the surgical stress and improved UES passage.

**Table 1:** Characteristics of aspiration prevention surgeries.

**Technique**	**Anesthesia**	Operation duration (min)^*^	Bleeding (mL)^**^	**UES relaxation**	**Voice prosthesis placement**
Total laryngectomy (13, 14, 23)	General > local	154–615	60–550	Improved	Possible
Central-part laryngectomy (9) (Case 1)	General	70–150	<50	Improved	Possible
Laryngotracheal diversion (14, 23, 24)	General or local	55–228	2–193	Unchanged	Dependent on cases
Laryngotracheal separation (11, 23, 25)	General or local	83–210	9–184	Unchanged	Dependent on cases
Laryngeal closure					
Glottic closure (7, 15) (Case 2)	Local > general	76–176	1–20	Unchanged	Impossible
Subglottic closure (SubC) (20, 26)	Local > general	85–290	10–120	Unchanged	Impossible
SubC with cricoidectomy (20) (Case 3)	General or local	125	Minor	Improved	Possible

* *The minimum to the maximum time*.

** *The minimum to the maximum bleeding amount. UES, upper esophageal sphincter*.

It is often the case that aspiration prevention surgeries deprive the patients of their voice, and substitute speech techniques, such as esophageal voice or artificial larynx, are recommended. However, prosthetic voice restoration after total laryngectomy has recently become possible. As it is considered that a voice prosthesis can be placed not only after total, but also after central-part, laryngectomy, and laryngeal closure with total cricoidectomy (Table 1), we will carefully observe the progress of case 1, with this treatment in mind, and will proceed when the patient requests it. Moreover, it should also be noted that pharyngeal flap surgery at the same time with aspiration prevention may improve patient quality of life when patients have concurrent velopharyngeal insufficiency resulting in inadequate pharyngeal pressure during swallowing.

## Conclusion

Dysphagia in neuromuscular disorders such as in Parkinson's disease, MSA, and ALS is caused by various mechanisms. Aspiration prevention for patients with neuromuscular disorders eliminates the possibility of aspiration pneumonia and airway obstruction and can prolong life expectancy. Appropriate choice of the surgical procedure for aspiration prevention under detailed preoperative examination, such as evaluation of UES relaxation during swallowing by HRMF, should be performed with consideration to patient physical status, expected time course, and the characteristics of the dysphagia.

## Data Availability Statement

The datasets generated for this study are available on request to the corresponding author.

## Ethics Statement

The studies involving human participants were reviewed and approved by the Human Ethics Committee of the University of Tokyo (No. 2487) and complied with the amended Declaration of Helsinki. Written informed consent was obtained from all patients in this study. The patients/participants provided their written informed consent to participate in this study.

## Author Contributions

MK was involved in patient care and surgery, collected information, and drafted the manuscript. RU conceived the study, was involved in patient care, surgery, follow-up, and preparation of the imaging, and drafted the manuscript. SS, TS, TG, and AY were involved in patient care and surgery and critically revised the manuscript. TY was involved in patient care and critically revised the manuscript.

### Conflict of Interest

The authors declare that the research was conducted in the absence of any commercial or financial relationships that could be construed as a potential conflict of interest.

## Abbreviations

ADPEC: Abnormal deglutitive proximal esophageal contraction
ALS: Amyotrophic lateral sclerosis
HRMF: High-resolution manofluorography
MSA: Multiple system atrophy
UES: Upper esophageal sphincter
VFSS: Videofluoroscopic swallowing study.
